# Neural Inhibition Task Elicits Age-Associated Changes in Prefrontal Cerebral Oxygenation

**DOI:** 10.1093/geroni/igaa057.1175

**Published:** 2020-12-16

**Authors:** Talia Salzman, Tabassum Rahman, Nadia Polskaia, Gabrielle St-Amant, Diana Tobón Vallejo, Yves Lajoie, Sarah Fraser

**Affiliations:** 1 University of Ottawa, Ottawa, Ontario, Canada; 2 University of Ottawa, Ottawa, Canada; 3 Universidad de Medellin, Medellin, Antioquia, Colombia

## Abstract

Certain cognitive tasks, such as those involving inhibition, can influence an older adult’s dual-tasking ability more than others. This study aimed to manipulate cognitive task difficulty to evaluate age-associated differences in brain activity and behaviour during walking. Nineteen younger (M=21.3, SD=3.9) and 20 older (M=71.8, SD=6.4) adults completed four cognitive-auditory tasks: simple reaction time (SRT; processing speed), Go-no-Go (GNG; neural inhibition), N-back (NBK; working memory) and Double number sequence (DNS; working memory) with or without self-paced walking. Trials took place under single cognitive (SC), single motor (SM) and dual-task (walking with a cognitive task; DT) conditions. Throughout each condition, cerebral oxygenation changes (ΔHbO2) in the prefrontal cortex were acquired using functional near-infrared spectroscopy (fNIRS). Behavioural measures including response time (ms), accuracy (%) and gait speed (m/s) were also calculated. Repeated measures ANOVAs revealed that OAs exhibited greater ΔHbO2 than YAs in the left hemisphere during the GNG inhibition task (p = 0.04). Activation in the right hemisphere also increased compared to the left during DNS DT (p = 0.05). Response times increased with increasing task difficulty and YAs were faster than OAs during NBK SC (p = 0.09). Neural findings revealed age-associated changes in prefrontal activation at the GNG inhibition difficulty level. Behavioural results indicated poorer performance with increasing task difficulty including slower response times in OAs. Moreover, gait speed and accuracy only decreased within task and difficulty. Therefore, understanding the neural and behavioural changes across task difficulty may help monitor cognitive decline and distinguish normal aging from disease states.

